# Does P-Cresylglucuronide Have the Same Impact on Mortality as Other Protein-Bound Uremic Toxins?

**DOI:** 10.1371/journal.pone.0067168

**Published:** 2013-06-24

**Authors:** Sophie Liabeuf, Griet Glorieux, Aurelie Lenglet, Momar Diouf, Eva Schepers, Lucie Desjardins, Gabriel Choukroun, Raymond Vanholder, Ziad A. Massy, on behalf of the European Uremic Toxin (EUTox) Work Group

**Affiliations:** 1 INSERM U-1088, Jules Verne University of Picardy, Amiens, France; 2 Clinical Research Centre - Division of Clinical Pharmacology, Amiens University Hospital and the Jules Verne University of Picardy, Amiens, France; 3 Nephrology Section, Department of Internal Medicine, University Hospital, Gent, Belgium; 4 Division of Nephrology-Dialysis-Transplantation, Amiens University Hospital, Amiens, France; 5 Division of Nephrology, Ambroise Paré Hospital, Paris-Boulogne Billancourt, France; Universidade de São Paulo, Brazil

## Abstract

**Background:**

Uremic toxins are emerging as important, non-traditional cardiovascular risk factors in chronic kidney disease (CKD). P-cresol has been defined as a prototype protein-bound uremic toxin. Conjugation of p-cresol creates p-cresylsulfate (PCS) as the main metabolite and p-cresylglucuronide (PCG), at a markedly lower concentration. The objective of the present study was to evaluate serum PCG levels, determine the latter’s association with mortality and establish whether the various protein-bound uremic toxins (i.e. PCS, PCG and indoxylsulfate (IS)) differed in their ability to predict mortality.

**Methodology/Principal Findings:**

We studied 139 patients (mean ± SD age: 67±12; males: 60%) at different CKD stages (34.5% at CKD stages 2–3, 33.5% at stage 4–5 and 32% at stage 5D). A recently developed high-performance liquid chromatography method was used to assay PCG concentrations. Total and free PCG levels increased with the severity of CKD. During the study period (mean duration: 779±185 days), 38 patients died. High free and total PCG levels were correlated with overall and cardiovascular mortality independently of well-known predictors of survival, such as age, vascular calcification, anemia, inflammation and (in predialysis patients) the estimated glomerular filtration rate. In the same cohort, free PCS levels and free IS levels were both correlated with mortality. Furthermore, the respective predictive powers of three Cox multivariate models (free PCS+other risk factors, free IS+other risk factors and free PCS+other risk factors) were quite similar - suggesting that an elevated PCG concentration has much the same impact on mortality as other uremic toxins (such as PCS or IS) do.

**Conclusions:**

Although PCG is the minor metabolite of p-cresol, our study is the first to reveal its association with mortality. Furthermore, the free fraction of PCG appears to have much the same predictive power for mortality as PCS and IS do.

## Introduction

Kidney failure is characterized by the gradual accumulation of several uremic retention compounds, some of which result from the degradation of proteins and amino acids [1]–[4]. When these compounds interact with biological functions, they are referred to as uremic toxins. In particular, protein-bound uremic compounds have recently received much attention [3], [5]. In the last decade, emerging data suggested that these solutes can cause several biological and physiologic alterations in a uremic setting [1], [6]. P-cresol (the precursor of p-cresylsulfate (PCS) and p-cresylglucuronide (PCG)) is mainly generated as an end product of tyrosine biotransformation by anaerobic intestinal bacteria [7], [8]. During passage through the colonic mucosa and liver, sulfatation and glucuronidation generates PCS (as the most preponderant metabolite) and PCG (at markedly lower concentrations) [9]. Various *in vitro* studies have shown that PCS causes endothelial damage and exerts a pro-inflammatory effect [6], [10] - in contrast to the mother compound p-cresol, which was shown to have an immunosuppressant effect [11]. However, to the best of our knowledge, the only data on the biological impact of PCG were reported recently by Meert et al [12]. The lack of studies on PCG may be due to its non-availability for purchase, the difficulty of its chemical preparation, the low presumed concentrations *in vivo* (based on indirect determinations) and the absence of a reliable determination method until recently [9]. On the basis of the *in vitro* experiments performed by Meert et al [12], it appears that PCG *per se* has no effect on leukocyte oxidative burst activity; however, when PCG was combined with PCS, a synergistic, cumulative leukocyte activating effect was observed [12].

We and others have demonstrated that total and free PCS serum levels (i) are elevated in late-stage CKD [13] and correlate with the glomerular filtration rate (GFR) in predialysis patients [14]. Free PCS was also found to be a predictor of survival in an analysis of both dialyzed and non-dialyzed CKD patients [14], [15]. We recently studied indoxylsulfate (IS, a protein-bound uremic toxin that results from the metabolism of dietary tryptophan) and found that it may be involved in the high incidence of vascular disease and mortality observed in CKD patients [16].

However, there are no published data on the respective impact of total and free PCG levels on outcomes in CKD patients. Even though PCG is a p-cresol conjugate with a relatively low total concentration, we decided to assess the clinical implications of the levels of this compound in CKD patients - especially since the free concentration (which is presumably the biologically active one) is quite similar to that of PCS [12]. Therefore, the objective of the present study was to evaluate the serum levels of PCG in a cohort of patients at different CKD stages. In addition, we sought to assess the link between PCG levels and overall and cardiovascular mortality. Lastly, since the impact of PCS and IS on mortality have already been evaluated in the same cohort, we compared the respective predictive powers of three different Cox models: free PCS+other risk factors, free IS+other risk factors and free PCG+other risk factors.

## Materials and Methods

### Ethics Statement

The study was conducted according to the principles of the Declaration of Helsinki and in compliance with International Conference on Harmonization/Good Clinical Practice regulations. Before the start of the study, patients were provided with comprehensive study information and all procedures were approved by a local investigational review board (*Comité de protection des Personnes Nord-Ouest II*, approval number 06H3) and the French healthcare authorities. All patients gave their written informed consent.

### Patient Selection

Over an 18-month period (from January 2006 to June 2007), a total of 150 Caucasian, prevalent CKD patients were recruited from the Nephrology Department’s outpatient clinic at Amiens University Hospital.

Included patients had to be over the age of 40, with a confirmed diagnosis of CKD (defined as being on hemodialysis or having two previous, estimated creatinine clearances (calculated according to the Cockcroft and Gault formula) <90 ml/min/1.73 m^2^, with an interval of 3 to 6 months). Stage 5D CKD patients had been on chronic hemodialysis three times a week for at least 3 months. The exclusion criteria consisted of the presence of chronic inflammatory disease, atrial fibrillation, complete heart block, abdominal aorta aneurysm, the presence of an aortic and/or femoral artery prosthesis, primary hyperparathyroidism, kidney transplantation and any acute cardiovascular event in the 3 months prior to screening for inclusion. The 139 patients who met all the inclusion criteria and none of the exclusion criteria and had available serum PCS and PCG quantification results were included in the present analysis.

### Study Protocol

All patients were hospitalized for the day in order to perform laboratory blood tests, blood pressure measurements, a pulse wave velocity (PWV) determination, a lateral lumbar X-ray and a multislice spiral computed tomography (MSCT) scan. For a given patient, all examinations were performed between 9am and 2pm on the same day. Hemodialysis patients were seen on a dialysis-free day or, if this was not possible, the morning before the dialysis session. A patient interview focused on comorbidities, the personal disease history and (in particular) any previous vascular events. The patients’ medical files were reviewed in order to identify and record any concomitant medications. For descriptive purposes, patients who reported current or past use of insulin and/or orally administered hypoglycemic drugs were considered to be diabetics. Previous cardiovascular disease was defined as a history of any of the following events: myocardial infarction, stroke, heart failure, angina pectoris, peripheral artery disease and any surgical procedure or percutaneous transluminal angioplasty because of vascular disease.

### Laboratory Tests

Blood samples were collected in the morning, before the other investigations were undertaken. Selected assays were performed after the samples had been frozen and stored at −80°C. Serum calcium, phosphate, albumin, cholesterol, hemoglobin, creatinine (Scr) and C-reactive protein levels were assayed in an on-site biochemistry laboratory using standard auto-analyzer techniques (the Modular IIP® system, Roche Diagnostics, Basel, Switzerland). Serum intact PTH (iPTH 1–84) was determined in a chemiluminometric immunoassay (Liaison N-tact PTH CLIA®, Diasorin, Stillwater, USA). The concentrations of PCS and PCG were determined with high-performance liquid chromatography (HPLC) as described recently using a Waters Alliance 2695 device (Waters, Zellik, Belgium) connected to a Waters 2475 fluorescence detector. To measure the total serum concentration, samples were diluted 1 to 6 with HPLC-grade water and then heated at 95°C for 30 minutes. After cooling (10 minutes on ice), samples were filtered through a Centrifree® filter (Millipore, Billerica, MA). The ultrafiltrate was injected onto Ultrasphere ODS column (150×4.6 mm, 5 µm particle size, Beckman Instruments, Fullerton, CA) with an Ultrasphere ODS guard column (45×4.6 mm, 5 µm article size, Beckman Instruments). To determine the free fraction, untreated serum samples were filtered through a Centrifree® prior to heating. Reference values for total PCS and free PCS in healthy subjects were 0.275±0.160 mg/dL and 0.008±0.009 mg/dL, respectively. Reference values for total PCG and free PCG in healthy subjects were 0.035±0.003 mg/dL and at the detection limit, respectively. To assay for serum IS, samples were deproteinized by heat denaturation and analyzed by reverse-phase, high-performance liquid chromatography. The serum concentrations were then determined by fluorescence detection (excitation: 280 nm; emission: 340 nm) using a reference value for healthy controls of 0.113±0.06 mg/100 mL. Serum cystatin C (CysC) levels were determined by immunonephelometry (N Latex Cystatin C®, Dade Behring, Marburg, Germany). In order to assess the true GFR in non-dialyzed patients as accurately as possible, the estimated GFR combining Scr and CysC measurements (CKD-epi) was calculated according to the following, recently published “CKD-epi” equation [17]: 177.6×Scr^−0.65^×CysC^−0.57^×age^−0.20^× (0.82 if female). For descriptive purposes, patients were then classified into CKD stages, according to the National Kidney Foundation’s Kidney Disease Outcomes Quality Initiative guidelines [18].

### Pulse Wave Velocity Evaluation

The carotid-femoral PWV was determined automatically with a dedicated device with two pressure probes (Complior Colson, Createch Industrie, Massy, France) and operated by a trained physician, as previously described [19]. Transcutaneously recorded pulse waveforms were obtained simultaneously for the common carotid artery and the femoral artery in the groin. The PWV was calculated as the distance between recording sites measured over the body’s surface (L), divided by the time interval (t) between the feet of the flow waves (PWV = L/t); this value was averaged over 10 cardiac cycles [20]. This automated method has been validated previously and has an intra-observer repeatability coefficient of 0.93 and an interobserver reproducibility coefficient of 0.89 [19], [20].

### Abdominal Aorta Imaging with Plain Radiography

A technique similar to that described by Kauppila et al. [21] was used to obtain images of the lower abdominal aorta and generate an aortic calcification score. All X-rays were reviewed by two independent investigators and a consensus on the interpretation was reached in all cases [22].

### Multislice Spiral Computed Tomography

In order to quantify the presence and extent of aortic calcifications, each patient underwent a MSCT scan. All examinations were performed with a 64-detector scanner (Lightspeed VCT®, GE Healthcare, Milwaukee, WI, USA).

The volume acquisition started at the aortic hiatus of the diaphragm and ended at the third lumbar vertebra. The scanning parameters were as follows: collimation: 64×0.625 mm; slice thickness: 0.625 mm; pitch: 1; gantry rotation speed: 0.5 s/rotation; tube voltage: 120 kV; tube current: 300 mA.

The volume acquisition was analyzed with commercially available software (Volume Viewer® software, GE Healthcare, Milwaukee, USA). The abdominal aorta was segmented manually. In order to reduce errors due to noise, a threshold of 160 UH was applied. The total calcification volume was calculated as the sum of all voxels in the remaining volume. The abdominal aorta calcification score was calculated as follows: [(total calcification volume)/(aorta wall surface area) * 100)].

### Survival

Death records were established prospectively, by considering all patients included at least twenty months before the study end date (March 1, 2009). Each medical chart was reviewed and the cause of death was assigned by a physician on the basis of all the available clinical information. For out-of-hospital deaths, the patient’s general practitioner was interviewed to obtain pertinent information on the cause. Cardiovascular mortality was defined as any death directly related to cardiovascular system dysfunction (stroke, myocardial infarction, congestive heart failure or sudden death).

### Statistical Analyses

Data are expressed as either the mean ± SD, the median or the frequency, as appropriate. Percent binding was calculated as total compound minus free compound divided by total compound *100. For descriptive and analytical purposes, the study population was stratified according the median serum free PCG concentration (i.e. serum free PCG ≤0.041 mg/dl vs. serum free PCG >0.041 mg/dl). Intergroup comparisons were performed using a χ^2^ test for categorical variables and Student’s *t* test or the Mann-Whitney test for continuous variables. For variables with a non-Gaussian distribution, log-normalized values were considered in tests that assumed normally distributed variables. A Kaplan-Meier actuarial curve was used to estimate overall and cardiovascular mortality. The log-rank test was used to compare the survival curves. Univariate and multivariate analyses of mortality were performed by using a Cox proportional hazards model of death as a function of free PCG levels as a continuous variable. In the multivariate analysis, the predefined models included variables found to be significantly associated with death in the univariate analyses (age, hemoglobin, CRP, aortic calcification score, eGFR and free PCG). As we did not use the backward selection option, we limited the number of variables for adjustment as a function of the number of events and created different models to include all the cited variables. Given that levels of the three uremic toxins evaluated here (PCS, PCG and IS) were highly intercorrelated (r^2^ = 0.95 free PCS and free PCG, r^2^ = 0.73 free PCG and free IS and r^2^ = 0.72 free IS and free PCS), we never included them all in the same model (i.e. to avoid colinearity bias). Hence, we used the Akaike information criterion (AIC) and the C-Index to evaluate the model’s power to predict mortality with each of the three uremic toxins in turn. Given that the respective impacts of PCS and IS on mortality have already been evaluated in the same cohort, we compared the predictive power of three different Cox models: free PCS+other risk factors, free IS+ other risk factors and free PCG+other risk factors. The predictive power of the three Cox models was assessed with Harrell’s C-index [23]. Harrell’s C index provides an estimate of the proportion of correct predictions, i.e., the proportion of patients with better staging and who had better survival. The C-index varies from 0.5 (no discrimination) to 1 (perfect discrimination).

A p value ≤0.05 was considered to be statistically significant. All statistical analyses were performed using SPSS software (SPSS Inc, Chicago, IL), version 13.0 for Windows (Microsoft Corp, Redmond, WA).

## Results

**Figure 1:**
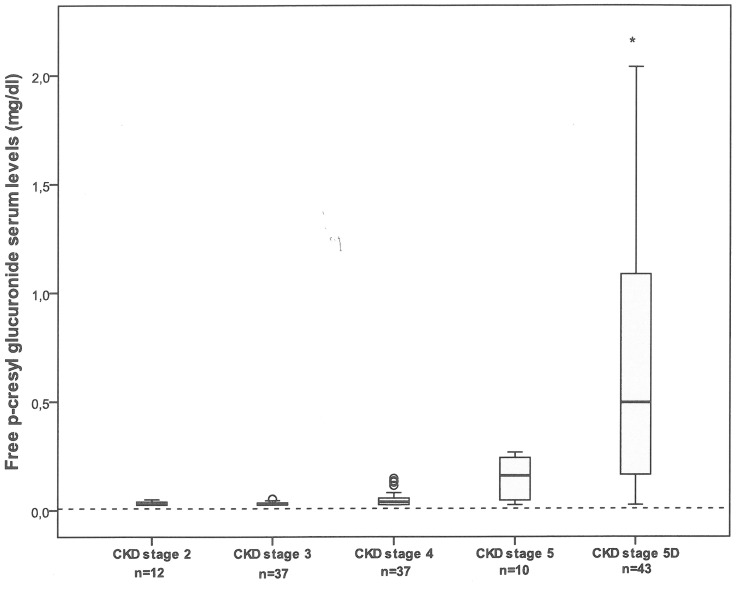
Serum levels of free p-cresylglucuronide as a function of CKD stage 2, 3, 4, 5 and 5D. *p<0.0001. The dotted line indicates the reference value for healthy subjects (0.035±0.003 mg/dL).

**Figure 2:**
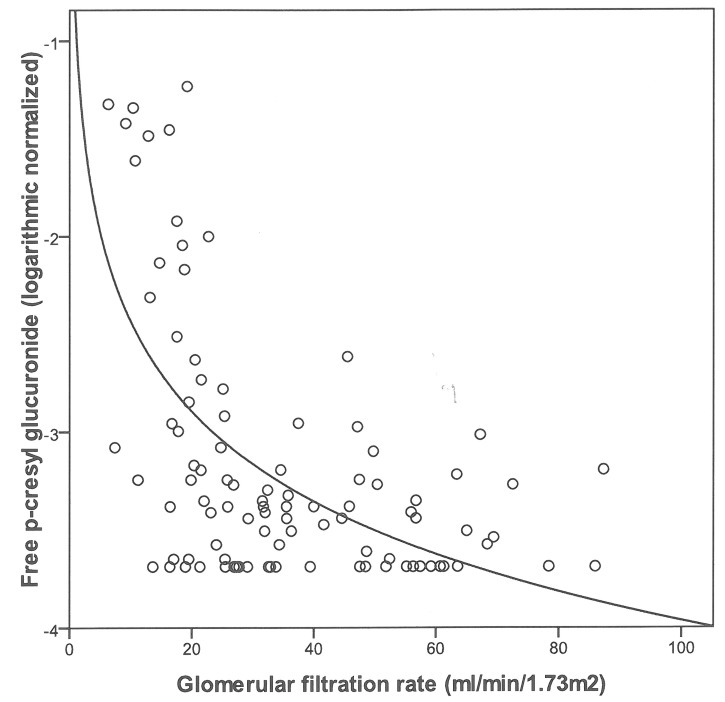
Logarithmic regression curves. The relationship between log-normalized free p-cresylglucuronide serum levels and the estimated glomerular filtration rate for patients at CKD stages 2 to 5; r^2^ = 0.34, p<0.0001 (n = 96).

**Figure 1** illustrates the distribution of free PCG levels by CKD stage. Serum levels of free PCG increase with the severity of CKD and were significantly higher in hemodialysis patients. When considering non-dialyzed patients only (n = 96), we observed a significant, inverse association between free PCG and the GFR, as illustrated in **Figure 2**. The same profile was found for total PCG (data not shown).

**Table 1** and **Table 2** depict the demographic, clinical and biochemical characteristics of the 139 analyzed patients according to the median free PCG level (total PCG ≤0.041 mg/dl vs. total PCG >0.041 mg/dl). The patients with higher free PCG serum levels had a lower body mass index and a more severe CKD stages. Concerning vascular parameters, patients with free PCG >0.041 mg/dl had a significantly higher aortic calcification score, whereas the two groups did not differ significantly in terms of PWV. Indeed, we found significant correlations between the aortic calcification score and free PCG (r = 0.30 p = 0.001) and between the coronary calcification score and free PCG (r = 0.24 p = 0.02). Regarding biochemical parameters, patients with a higher free PCG had higher serum phosphate, iPTH, free PCS, free IS, total PCG, percentage PCG protein binding, lower hemoglobin levels and GFR-epi. There were no differences in terms of total PCG or free PCG when we divided the population according to diabetes status (n = 59 diabetic patients).

**Table 1:** Clinical and demographic characteristics of the study population.

		Free p-cresylglucuronide		
	All n = 139	≤0.041 mg/dL n = 70	>0.041 mg/dL n = 69	p
Age, years	67±12	68±12	66±13	0.5
Male gender, n (%)	84 (60)	45 (63)	39 (57)	0.5
Body mass index (kg/m^2^)	28±6	29±6	27±6	0.02
Diabetes mellitus, n (%)	59 (42)	31 (44)	28 (41)	0.9
Smoking habit, n (%)	56 (41)	29 (41)	27 (39)	0.9
Presence of CVD, n (%)	43 (31)	18 (26)	25 (36)	0.1
Systolic arterial pressure (mmHg)	153±26	152±24	156±29	0.4
Diastolic arterial pressure (mmHg)	81±12	82±11	81±13	0.9
Lipid-lowering therapy n (%)	83 (60)	46 (65)	37 (56)	0.2
Antihypertensive drugs n (%)	125 (90)	66 (94)	59 (85)	0.2
CKD stage, n (%)				<0.0001
2	12 (8)	11 (15)	1 (17)	
3	37 (26.5)	33 (46)	4 (7)	
4	37 (26.5)	21 (30)	16 (23)	
5	10 (7)	2 (17)	8 (12)	
5D	43 (32)	5 (7)	38 (55)	
Aortic calcificationscore on CT,%	3.0±3.0 (1.9)	2.3±2.5 (1.4)	3.6±3.3 (2.6)	0.01
Aortic calcificationscore on X-ray, scale 0–24	6.3±6.6 (4.5)	4.5±5.8 (3.0)	7.6±6.9 (6.0)	0.008
PWV, m/s	14.8±3.8	14.4±3.6	14.7±4	0.6

Data are expressed as means ± SD and (median) for variables with a non-Gaussian distribution or, for binary variables, the number (frequency). CVD: cardiovascular disease; CKD: chronic kidney disease; CT: computed tomography; PWV: pulse wave velocity.

**Table 2:** Biochemical characteristics of the study population.

		Free p-cresylglucuronide		p value
	All n = 139	≤0.041 mg/dL n = 70	>0.041 mg/dL n = 69	All n = 139
Calcium, mMol/L	2.3±0.18	2.3±0.14	2.3±0.22	0.4
Phosphate, mMol/L	1.3±0.46	1.2±0.37	1.4±0.51	0.005
Intact PTH, pg/mL	137±138 (85)	102±99 (65)	175±162 (121)	<0.0001
Albumin, g/L	38±6	38±7	37±6	0.1
C-reactive protein, mg/L	10.7±23 (3.5)	8.9±16 (2.5)	14±30 (4.0)	0.2
Hemoglobin, g/dL	12.0±1.7	12.7±1.7	11.5±1.6	<0.0001
GFR-epi§, mL/min/1.73 m^2^	35±19	41±18	23±14	<0.0001
Total cholesterol, mMol/L	4.9±1.2	5.0±1.1	4.8±1.2	0.2
LDL cholesterol, mMol/L	2.7±0.9	2.7±0.9	2.6±0.9	0.4
Triglycerides, mMol/L	2.0±1.3	2.0±1.5	2.0±1.1	0.7
Free p-cresylsulfate, mg/dL	0.26±0.51 (0.051)	0.02±0.02 (0.02)	0.51±0.64 (0.26)	<0.0001
Free indoxylsulfate, mg/dl	0.08±0.09 (0.038)	0.04±0.01 (0.037)	0.13±0.12 (0.08)	<0.0001
Free p-cresylglucuronide, mg/dL	0.27±0.54 (0.041)	0.03±0.005 (0.028)	0.53±0.69 (0.22)	NA
Total p-cresylglucuronide, mg/dL	0.29±0.54 (0.046)	0.03±0.008 (0.03)	0.58±0.75 (0.25)	<0.0001
p-cresylglucuronide, % binding	9.3±7.3 (8.1)	7.3±6.9 (6.9)	11.5±7 (10.6)	<0.0001

Data are expressed as means ± SD and (median) for variables with a non-Gaussian distribution. PTH: parathyroid hormone; GFR: glomerular filtration rate; LDL: low-density lipoprotein.

§ calculated for patients at CKD stages 2 to 5 (n = 96).

**Figure 3:**
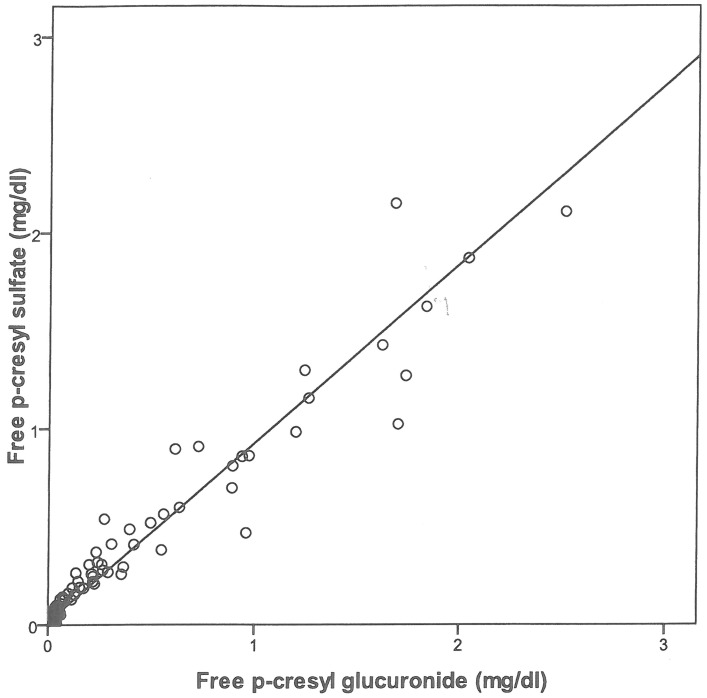
Linear regression curves showing the relationship between free p-cresylglucuronide serum levels and free p-cresylsulfate serum levels; r2 = 0.95, p<0.0001.

**Table 3** compares the serum levels of PCS and PCG; the total mean concentration of PCS is substantially higher than that of PCG, whereas the free levels are similar. The conjugates differed markedly in terms of the percentage bound to protein (91.4±10.9% for PCS vs. only 9.3±7.3% for PCG). Furthermore, the respective levels of PCS and PCG were highly correlated, with r^2^ = 0.95 for the free fraction (**Figure 3**) and r^2^ = 0.51 for the total concentration (data not shown).

**Table 3:** Serum concentrations of p-cresylglucuronide and p-cresylsulfate (total, free and bound) in the study population.

	p-cresylglucuronide	p-cresylsulfate
**total (mg/dl)**	0.29±0.58	1.94±1.84
**free (mg/dl)**	0.27±0.54	0.26±0.51
**bound (mg/dl)**	0.03±0.05	1.64±1.39
**% protein bound**	9.30±7.3	91.40±10.96

**Figure 4:**
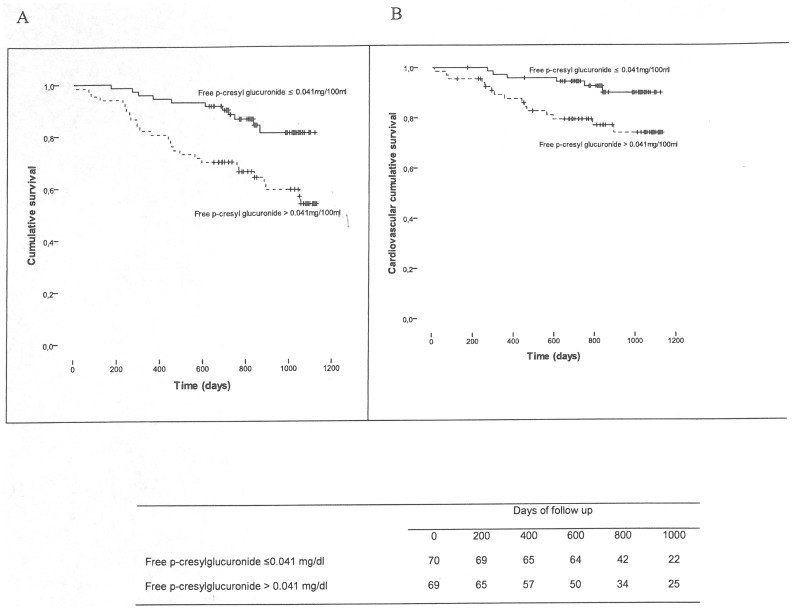
Mortality and free p-cresylglucuronide levels (A) Kaplan–Meier estimates of overall mortality as a function of free p-cresylglucuronide levels relative to the median; p = 0.002 in a log-rank comparison of the curves. (B) Kaplan–Meier estimates of cardiovascular mortality as a function of free p-cresylglucuronide levels relative to the median; p = 0.01 in a log-rank comparison of the curves.

During the study period (mean follow-up duration: 779±185 days; median (range): 815 (10–1129), 38 patients died (22 from CV causes, 8 from infectious disease and 8 from other causes). In a crude analysis (**Figure 4**), free PCG >0.041 mg/dl was a significant predictor of overall and cardiovascular death (log-rank comparison of the curves: p = 0.002 and p = 0.01 respectively).

In a univariate Cox regression analysis, free IS, free PCS, total PCG, free PCG, age, dialysis status, albumin, hemoglobin, C-reactive protein and the aortic calcification score and eGFR (for predialysis patients) were all significantly associated with the risk of death (data not shown).

**Figure 5:**
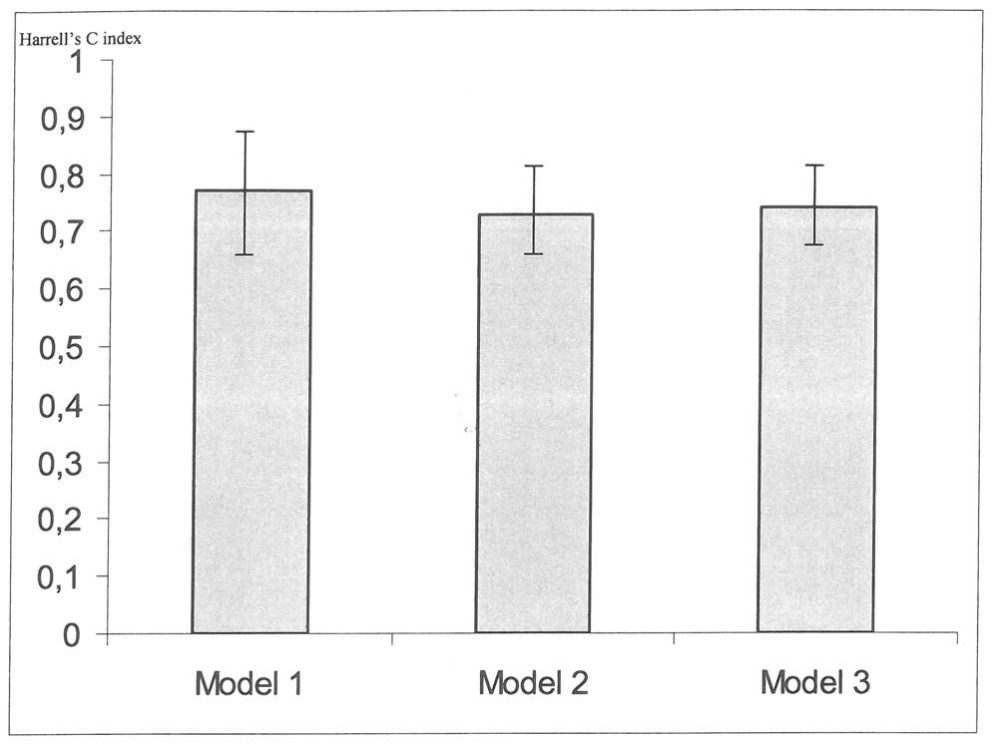
Harrell’s C index according to the Cox models (Model 1: Ln free PCS+aortic calcification+CRP+Hb; Model 2: Ln free IS +aortic calcification+CRP+Hb; Model 3: Ln free PCG +aortic calcification+CRP+Hb).

**Table 4** shows the predictive power of free PCG levels for death in unadjusted models or models adjusted for multiple covariates. After adjusting the different models for age, hemoglobin, C-reactive protein and the aortic calcification score, higher serum levels of free PCG still had a significant effect on the risk of death. It is noteworthy that high levels of free PCG were also independently associated with cardiovascular mortality, as shown in Table 5. These results were confirmed when the crude analysis was restricted to CKD pre-dialysis patients (n = 96, deaths = 18) and after adjustment for the same covariables and eGFR (**Table 6**). The univariate Cox model’s ability to predict the mortality associated with each of the three uremic toxins was estimated by calculation of the Akaike information criterion (AIC), with values of 350, 349 and 352 for PCS, PCG and IS, respectively. In addition, **Figure 5** shows Harrell’s C index for three multivariate models including aortic calcification score, CRP, hemoglobin and, respectively, free PCS, free IS and free PCG. Harrell’s C index [95%CI] was 0.77 [0.68–0.95] for the free PCS model; 0.73 [0.64–0.82] for the free IS model and 0.74 [0.64–0.83] for the free PCG model). The similar values of Harrell’s C index suggest that the three variables do not differ significantly in terms of predictive performance.

**Table 4:** Univariate and multivariate Cox regression analysis of risk factors at baseline for all-cause mortality, with free p-cresylglucuronide entered as a continuous variable in all patients (n = 139).

Models of patient survival (event n = 38)	RR	95% CI	p
Unadjusted			
Ln free p-cresylglucuronide	1.335	1.095–1.628	0.004
Model adjusted for age and hemoglobin			
Ln free p-cresylglucuronide	1.32	1.05–1.64	0.015
Age	1.05	1.02–1.08	0.003
Hemoglobin	0.74	0.61–0.89	0.002
Model adjusted for age and CRP			
Ln free p-cresylglucuronide	1.42	1.15–1.76	0.001
Age	1.05	1.02–1.09	0.002
CRP	1.007	0.99–1.02	0.073
Model adjusted for age and the aortic calcification score			
Ln free p-cresylglucuronide	1.37	1.09–1.72	0.007
Age	1.04	0.99–1.08	0.07
Aortic calcification score	1.15	1.03–1.29	0.01

RR: risk ratio; CI: confidence interval.

**Table 5:** Univariate and multivariate Cox regression analyses of risk factors at baseline for cardiovascular mortality, with free p-cresylglucuronide entered as a continuous variable in all patients (n = 139).

Models of patient survival (event n = 22)	RR	95% CI	p
Unadjusted			
Ln free p-cresylglucuronide	1.85	1.08–1.61	0.003
Model adjusted for age*			
Ln free p-cresylglucuronide	1.80	1.01–3.21	0.046
Model adjusted for hemoglobin**			
Ln free p-cresylglucuronide	1.98	1.11–3.52	0.021
Model adjusted for CRP			
Ln free p-cresylglucuronide	2.24	1.22–4.10	0.0009
CRP	1.02	1.00–1.05	0.038
Model adjusted for the aortic calcification score			
Ln free p-cresylglucuronide	1.96	1.06–3.61	0.031
Aortic calcification score	1.21	1.02–1.42	0.026

RR: risk ratio; CI: confidence interval.

* age was not significant after adjustment.

** hemoglobin was not significant after adjustment.

**Table 6:** Univariate and multivariate Cox regression analysis of risk factors at baseline for all-cause mortality, with free p-cresylglucuronide entered as a continuous variable in the predialysis population (n = 96).

Models of patient survival (event n = 18)	RR	95% CI	p
Unadjusted			
Ln free p-cresylglucuronide	1.98	1.11–3.53	0.021
Model adjusted for age			
Ln free p-cresylglucuronide	1.81	1.01–3.21	0.04
Age	1.05	1.00–1.09	0.05
Model adjusted for hemoglobin*			
Ln free p-cresylglucuronide	1.78	1.01–3.21	0.04
Model adjusted for CRP			
Ln free p-cresylglucuronide	2.20	1.20–4.02	0.01
CRP	1.02	1.00–1.05	0.05
Model adjusted for the aortic calcification score			
Ln free p-cresylglucuronide	1.89	1.01–3.50	0.04
Aortic calcification score	1.6	1.01–2.61	0.04
Model adjusted for eGF **			
Ln free p-cresylglucuronide	1.98	1.11–3.51	0.02

RR: risk ratio; CI: confidence interval.

* hemoglobin was not significant after adjustment.

** eGFR was not significant after adjustment.

## Discussion

Our study demonstrated that both total and free PCG levels were rose with the decrease of GFR and are increased in hemodialysis patients. More importantly, free and total PCG levels were associated with total and cardiovascular mortality. Moreover, it appears that the three uremic toxins investigated (PCS, PCG and IS) were able to predict mortality in the present cohort to the same extent.

Firstly, in a large cohort of patients at various CKD stages, we confirmed that the serum concentration of PCG is elevated, as also recently reported [12] in a smaller group of uremic dialysis patients. We also found an inverse relationship between free/total PCG levels and the glomerular filtration rate, with toxin accumulation greatest in patients on dialysis. It has been demonstrated that conjugation of p-cresol results in high concentrations of PCS (as the main metabolite) and markedly lower concentrations of total PCG [9], [12]. We confirmed this finding, as levels of total PCS were approximately 7-fold higher than those of total PCG in our cohort (as shown in Table 3). However, the conjugates showed a marked difference in percentage binding to protein, with high values for PCS (91.4±10.9%) and low ones for PCG (9.3±7.3%). However, although most of PCS is bound whereas PCG to the largest extent is free, their absolute free concentrations appear to be identical. Our present results differ from those of Bergé-Lefranc et al., who reported substantially higher free PCS levels. However, the latter authors used isothermal titration calorimetry to assay for PCS; this methodological difference may explain the disparities in the results [24]. However, Itoh et al. [25] recently used a newly developed method (involving simultaneous LC/ESI-MS/MS) to determine levels of PCS binding that were similar to those observed here, whereas PCG binding was higher than in the present study.

Secondly, we demonstrated that serum PCG levels were associated with increased aortic calcification in the study cohort, since we observed a positive correlation between serum PCG levels and the aortic/coronary calcification scores. Recent research has revealed how some uremic toxins (including PCS and IS) can produce toxic biological effects. However, to the best of our knowledge, the impact of PCG on vascular calcification has not yet been evaluated. Recently published data show that indoxylglucuronides (such as IS) can induce reactive oxygen species production in human umbilical vein endothelial cells [25]. Furthermore, oxidative stress has been linked to vascular calcification [26]. We are now actively working to investigate this issue.

Lastly, we have shown for the first time that higher free and total PCG concentrations were associated with mortality, independently of well-known predictors of survival (such as age, vascular calcification, anemia, inflammation and eGFR for predialysis patients). These results are in line with previous reports on the mother compound, p-cresol [27], [28]. Overall, studies in CKD cohorts have found a negative impact of free (but not total) p-cresol/PCS on the relative risk of mortality [14], [28]–[30]. In contrast to the findings for p-cresol/PCS, we demonstrated that the clinical impact of PCG is relevant for both free and total fractions. The protein-bound fraction of PCG is low so that free and total concentrations are much closer to each other than is the case for PCS. Therefore, one can legitimately assume that the total and free PCG fractions should have similar weightings in outcome studies. It is noteworthy than in our cohort of patients at different stages of CKD, the free fractions of the three protein-bound uremic toxins (PCS, PCG and IS) did not differ significantly in terms of their respective abilities to predict mortality. Hence, these uremic toxins may be potential therapeutic targets for preventing renal complication. Indeed, the AST-120 oral charcoal adsorbent (Kremezin, Kureha Chemical Industry, Tokyo, Japan) adsorbs hydrophobic uremic toxins and has shown promise in animal studies and epidemiologic surveys. Indeed, AST 120 has been shown to attenuate the reduction in estimated creatinine clearance in CKD patients [31] and improve uremic syndrome [32]. Beneficial impacts on cardiovascular and bone complications have also been suggested [33]–[34]. These beneficial effects has been attributed to its action on IS and PCS levels, but potential effects on PCG although possible did not yet explored.

The present study’s limitations include the relatively small sample size and the small number of protein-bound uremic toxins assayed simultaneously. However, PCS and IS are the most frequently studied compounds within this group of uremic toxins. In contrast, the study’s main strengths are (i) the inclusion of patients at different CKD stages, (ii) the application of a novel analytical method for assaying both the major and the minor conjugate of p-cresol (since data on the latter had been lacking until now).

In conclusion, this is the first study to have evaluated free and total levels of PCG – a hitherto neglected *in vivo* metabolite of p-cresol - in patients at different stages of CKD. Our data emphasize the significance of free and total PCG as predictors of survival, despite the fact that the compound is only a minor metabolite of p-cresol. However, future research must further elucidate the role of PCG and confirm that uremic toxins that can bind to protein are interchangeable biomarkers of mortality.

## References

[1] Vanholder R, De Smet R (1999) Pathophysiologic effects of uremic retention solutes. J Am Soc Nephrol. Aug 10(8): 1815–23. 10.1681/ASN.V1081815 10446951

[2] Vanholder R, De Smet R, Hsu C, Vogeleere P, Ringoir S (1994) Uremic toxicity: the middle molecule hypothesis revisited. Semin Nephrol. May 14(3): 205–18. 8036355

[3] Vanholder R, Van Laecke S, Glorieux G (2008) What is new in uremic toxicity? Pediatr Nephrol. Aug 23(8): 1211–21. 10.1007/s00467-008-0762-9 PMC2441592 18324423

[4] Vanholder R, Baurmeister U, Brunet P, Cohen G, Glorieux G, et al (2008) A bench to bedside view of uremic toxins. J Am Soc Nephrol. May 19(5): 863–70. 10.1681/ASN.2007121377 18287557

[5] Jourde-Chiche N, Dou L, Cerini C, Dignat-George F, Brunet P (2011) Vascular incompetence in dialysis patients–protein-bound uremic toxins and endothelial dysfunction. Semin Dial. May-Jun 24(3): 327–37. 10.1111/j.1525-139X.2011.00925.x 21682773

[6] Schepers E, Meert N, Glorieux G, Goeman J, Van der Eycken J, et al (2007) P-cresylsulphate, the main in vivo metabolite of p-cresol, activates leucocyte free radical production. Nephrol Dial Transplant. Feb 22(2): 592–6. 10.1093/ndt/gfl584 17040995

[7] Curtius HC, Mettler M, Ettlinger L (1976) Study of the intestinal tyrosine metabolism using stable isotopes and gas chromatography-mass spectrometry. J Chromatogr. Nov 3 126: 569–80. 10.1016/s0021-9673(01)84102-9 977696

[8] De Smet R, Van Kaer J, Van Vlem B, De Cubber A, Brunet P, et al (2003) Toxicity of free p-cresol: a prospective and cross-sectional analysis. Clin Chem. Mar 49(3): 470–8. 10.1373/49.3.470 12600960

[9] De Loor H, Bammens B, Evenepoel P, De Preter V, Verbeke K (2005) Gas chromatographic-mass spectrometric analysis for measurement of p-cresol and its conjugated metabolites in uremic and normal serum. Clin Chem. Aug 51(8): 1535–8. 10.1373/clinchem.2005.050781 16040852

[10] Meijers BK, Van Kerckhoven S, Verbeke K, Dehaen W, Vanrenterghem Y, et al. (2009) The Uremic Retention Solute p-Cresyl Sulfate and Markers of Endothelial Damage. Am J Kidney Dis. Jul 16. 10.1053/j.ajkd.2009.04.022 19615803

[11] Dou L, Cerini C, Brunet P, Guilianelli C, Moal V, et al (2002) P-cresol, a uremic toxin, decreases endothelial cell response to inflammatory cytokines. Kidney Int. Dec 62(6): 1999–2009. 10.1046/j.1523-1755.2002.t01-1-00651.x 12427124

[12] Meert N, Schepers E, Glorieux G, Van Landschoot M, Vanholder R (2012) Novel method for simultaneous determination of p-cresylsulfate and p-cresylglucuronide : clinical data and pathophysiological implications Nephrol Dial Transplant. Jun 27(6): 2388–96. 10.1093/ndt/gfr672 22167586

[13] Niwa T, Tsukushi S, Ise M, Miyazaki T, Tsubakihara Y, et al (1997) Indoxyl sulfate and progression of renal failure: effects of a low-protein diet and oral sorbent on indoxyl sulfate production in uremic rats and undialyzed uremic patients. Miner Electrolyte Metab. 23(3–6): 179–84. 9387112

[14] Liabeuf S, Barreto DV, Barreto FC, Meert N, Glorieux G, et al (2010) Free p-cresylsulphate is a predictor of mortality in patients at different stages of chronic kidney disease. Nephrol Dial Transplant. Apr 25(4): 1183–91. 10.1093/ndt/gfp592 19914995

[15] Wu IW, Hsu KH, Lee CC, Sun CY, Hsu HJ, et al (2011) p-Cresyl sulphate and indoxyl sulphate predict progression of chronic kidney disease. Nephrol Dial Transplant. Mar 26(3): 938–47. 10.1093/ndt/gfq580 PMC3042976 20884620

[16] Barreto FC, Barreto DV, Liabeuf S, Meert N, Temmar M, et al (2009) Serum indoxyl sulfate is associated with vascular disease and mortality in chronic kidney disease patients. Clin J Am Soc Nephrol. Oct 4(10): 1551–8. 10.2215/CJN.03980609 PMC2758258 19696217

[17] Stevens LA, Coresh J, Schmid CH, Feldman HI, Froissart M, et al (2008) Estimating GFR using serum cystatin C alone and in combination with serum creatinine: a pooled analysis of 3,418 individuals with CKD. Am J Kidney Dis. Mar 51(3): 395–406. 10.1053/j.ajkd.2007.11.018 PMC2390827 18295055

[18] K/DOQI (2002) Clinical practice guidelines for chronic kidney disease: evaluation, classification, and stratification. Am J Kidney Dis. Feb 39(2 Suppl 1)S1–266. 11904577

[19] Zureik M, Temmar M, Adamopoulos C, Bureau JM, Courbon D, et al (2002) Carotid plaques, but not common carotid intima-media thickness, are independently associated with aortic stiffness. J Hypertens. Jan 20(1): 85–93. 10.1097/00004872-200201000-00013 11791030

[20] Asmar R, Benetos A, Topouchian J, Laurent P, Pannier B, et al (1995) Assessment of arterial distensibility by automatic pulse wave velocity measurement. Validation and clinical application studies. Hypertension. Sep 26(3): 485–90. 10.1161/01.hyp.26.3.485 7649586

[21] Kauppila LI, Polak JF, Cupples LA, Hannan MT, Kiel DP, et al (1997) New indices to classify location, severity and progression of calcific lesions in the abdominal aorta: a 25-year follow-up study. Atherosclerosis. Jul 25 132(2): 245–50. 10.1016/s0021-9150(97)00106-8 9242971

[22] Temmar M, Liabeuf S, Renard C, Czernichow S, Esper NE, et al (2010) Pulse wave velocity and vascular calcification at different stages of chronic kidney disease. J Hypertens Jan 28(1): 163–9. 10.1097/HJH.0b013e328331b81e 19927012

[23] Harrell FE, Lee KL, Mark DB (1996) Tutorial in biostatistics. Multivariable prognostic models issues in developing models, evaluating assumptions and adequacy, and measuring and reducing errors. Statist Med. 15: 361–387. 10.1002/(SICI)1097-0258(19960229)15:4<361::AID-SIM168>3.0.CO;2-4 8668867

[24] Berge-Lefranc D, Chaspoul F, Calaf R, Charpiot P, Brunet P, et al (2010) Binding of p-cresylsulfate and p-cresol to human serum albumin studied by microcalorimetry. J Phys Chem B. Feb 4 114(4): 1661–5. 10.1021/jp9059517 20067224

[25] Itoh Y, Ezawa A, Kikuchi K, Tsuruta Y, Niwa T (2012) Protein-bound uremic toxins in hemodialysis patients measured by liquid chromatography/tandem mass spectrometry and their effects on endothelial ROS production. Anal Bioanal Chem. Jun 403(7): 1841–50. 10.1007/s00216-012-5929-3 22447217

[26] Massy ZA, Stenvinkel P, Drueke TB (2009) The role of oxidative stress in chronic kidney disease. Semin Dial. Jul-Aug 22(4): 405–8. 10.1111/j.1525-139X.2009.00590.x 19708991

[27] Lin CJ, Wu CJ, Pan CF, Chen YC, Sun FJ, et al (2010) Serum protein-bound uraemic toxins and clinical outcomes in haemodialysis patients. Nephrol Dial Transplant. Nov 25(11): 3693–700. 10.1093/ndt/gfq251 20466687

[28] Meijers BK, Claes K, Bammens B, de Loor H, Viaene L, et al (2010) p-Cresol and cardiovascular risk in mild-to-moderate kidney disease. Clin J Am Soc Nephrol. Jul 5(7): 1182–9. 10.2215/CJN.07971109 PMC2893077 20430946

[29] Liabeuf S, Drueke T, Massy Z (2011) Protein-Bound uremic toxins : new insight from clinical studies. Toxins. 3: 911–9. 10.3390/toxins3070911 PMC3202851 22069747

[30] Meijers BK, Bammens B, De Moor B, Verbeke K, Vanrenterghem Y, et al (2008) Free p-cresol is associated with cardiovascular disease in hemodialysis patients. Kidney Int. May 73(10): 1174–80. 10.1038/ki.2008.31 18305466

[31] Akizawa T, Asano Y, Morita S, Wakita T, Onishi Y, et al (2009) Effect of a carbonaceous oral adsorbent on the progression of CKD: a multicenter, randomized, controlled trial. Am J Kidney Dis 54: 459–67. 19615804 10.1053/j.ajkd.2009.05.011

[32] Schulman G, Agarwal R, Acharya M, Berl T, Blumenthal S, et al (2006) Multicenter, randomized, double-blind, placebocontrolled, dose-ranging study of AST-120 (Kremezin) in patients with moderate to severe CKD. Am J Kidney Dis 47: 565–77. 16564934 10.1053/j.ajkd.2005.12.036

[33] Nakamura T, Kawagoe Y, Matsuda T, Ueda Y, Shimada, et al (2004) Oral adsorbent AST-120 decreases carotid intima-media thickness and arterial stiffness in patients with chronic renal failure. Kidney Blood Press Res 27: 121–6. 15051932 10.1159/000077536

[34] Iwasaki Y, Yamato H, Nii-Kono T, Fujieda A, Uchida M, et al (2006) Administration of oral charcoal adsorbent (AST-120) suppresses low-turnover bone progression in uraemic rats. Nephrol Dial Transplant 21: 2768–74. 16820376 10.1093/ndt/gfl311

